# Hypothesis: Taurine therapy of nephropathic cystinosis may correct the deficiencies of cysteamine therapy

**DOI:** 10.1016/j.ymgmr.2025.101236

**Published:** 2025-06-11

**Authors:** Jess G. Thoene

**Affiliations:** Department of Pediatrics, Division of Pediatric Genetics, Metabolism & Genomic Medicine, University of Michigan, Ann Arbor, MI, United States of America

**Keywords:** Cystinosis, Fanconi syndrome, Taurine, Autophagocytosis, Apoptosis

## Abstract

Untreated nephropathic cystinosis is a lethal autosomal recessive disease. The current specific therapy, cysteamine, ameliorates the renal function loss, but does not alter the renal Fanconi syndrome, short stature, muscle weakness, male infertility, and other concerns. The primary biochemical/physiological defect in cystinosis is failure to supply cysteine to mTOR via cystinosin. This leads mTOR to react in starvation mode, which stops cell differentiation, leading to proximal tubule loss, and ultimately renal failure. It also increases apoptosis and autophagocytosis rates, which may contribute to impaired growth. Many of the defects which occur in cystinosis are corrected by taurine in other conditions as described. Cystinosis patients have been shown to be severely deficient in plasma taurine. Although use of taurine is not yet reported in cystinosis *in vitro* or *in vivo*, given the safety of taurine, its deficiency in cystinosis, and its potency in correcting similar defects in other conditions, it appears reasonable to engage in a clinical trial of taurine in nephropathic cystinosis.

## 1 Cystinosis: description, detrimental effects of CTNS mutation on mTOR, cysteamine failures

Cystinosis has been known as a clinical entity since early in the 20th century. Abderhalden first described two children in a family who died of inanition, and who had tiny white crystals in their liver [1]. Subsequently nephropathic cystinosis has been described with increasing detail which includes the renal Fanconi syndrome, and renal failure by about 10 years of age. Other significant clinical findings include short stature, never reaching greater than the 3rd percentile for age, hypercholesterolemia, pulmonary complications, metabolic bone disease, diabetes, cutaneous abnormalities, vascular calcifications, progressive muscle weakness, eye findings, including corneal crystals, and pigmentary retinopathy [1].

Symptomatic treatment of nephropathic cystinosis has improved over the ensuing century, but was initially limited to replacement of electrolytes and water to reduce the danger of electrolyte imbalance and dehydration. Additionally, it was found that the children develop vitamin D responsive rickets. However, no specific therapy was available to treat the underlying defect, until, in the 1970s, it was found that cystine accumulates in lysosomes of cystinotic cells [1]. In 1976, cysteamine was found to cause cystine depletion in vitro and in vivo [2,3]. Cysteamine reacts inside lysosomes in cystinotic tissues to form a new compound with cystine called cysteamine-cysteine mixed disulfide, which exits lysosomes on the intact lysine transporter [4]. The reason for the lysosomal cystine accumulation was unknown until the 1980s when it was found that the lysosomal transporter for cystine (CTNS) is mutated in cystinosis leading to an increase of cystine in lysosomes about 100-fold above normal [1].

Oral systemic cysteamine therapy for nephropathic cystinosis was subsequently approved by the US FDA in 1994. It did not prevent renal failure, but improved growth, and delayed the need for renal transplantation by about 10 years [5].

Many clinical issues are not resolved by systemic cysteamine treatment of nephropathic cystinosis. These include the renal Fanconi syndrome, which is one of the most persistent and potentially debilitating elements of the cystinotic phenotype and, although it may be partially corrected by cysteamine. Massive polyuria and failure to reabsorb electrolytes and other essential small molecules remain and cause the children to be at risk for serious dehydration and electrolyte imbalance [1]. Other adverse phenotypic developments not responsive to systemic cysteamine therapy include corneal crystals, inflammation, short stature, hypercholesterolemia, premature skin aging, metabolic bone disease, male infertility, vascular calcifications, muscle weakness, pulmonopathy, and diabetes (Table 1). Some of the abnormalities develop in adolescence or adulthood [5,6].

**Table 1:** Clinical elements in the nephropathic cystinosis phenotype uncorrected by systemic cysteamine.

Renal Fanconi Syndrome
Inflammation
Corneal crystals
Retinopathy
Delayed renal failure (not averted)
Short stature
Hypercholesterolemia
Male infertility
Elements premature aging • Skin • Vascular calcifications • Bone changes • Muscle weakness • Pulmonary complications Diabetes • Hypothyroidism

In addition to the clinical abnormalities, nephropathic cystinosis displays abnormalities in cell physiology which contribute to the clinical phenotype (Table 2). These abnormalities center around mTOR, a serine/threonine kinase and major regulator of cell metabolic activity [7,8]. Cystinosin had previously been described as interacting with mTOR [9,10], but its centrality in cystinosis was noted in the Berquez paper [11] in which it was shown that proximal tubule (PT) cells in the kidney degrade disulfide containing proteins after endocytosis, generating cystine. Defective cystine transport from lysosomes due to mutated cystinosin stimulates Ragulator-Rag GTPase-recruitment of the mechanistic target of rapamycin complex 1 leading to its constitutive activation [11] and causing a shift away from cell specialization. It was already well established that cystinotic cells accumulate cystine from the lysosomal degradation of cystine-rich proteins [12,13], and that atubular glomeruli occur as proximal renal tubules are lost. In a study of cystinosis and normal renal biopsies, it was found that cystinotic kidneys at end stage had 69 % atubular glomeruli and 30 % atrophic glomeruli. Normal renal tissue had 4 % atubular glomeruli and 0 % atrophic glomeruli (*p* < 0.0001 for both comparisons [14]. This progression leads to global loss of renal filtration function [5].

**Table 2:** Elements in cystinosis cell pathology leading to clinical phenotypes.

Loss of cell specialization • Create Renal Fanconi Syndrome
Increased apoptosis
Increased autophagy
Aberrant intermediate metabolism • Lipid synthesis • Nucleotide synthesis • Biogenesis of lysosomes • Nutrient sensing • Growth factor signaling
Aberrant osteocyte development
Aberrant myocyte development
Cystine crystal inflammatory processes

Thus it isn't cystine storage that does the damage in cystinosis, but deficiency of available cytosolic cysteine. Interestingly, neither extracellular cysteine, *N*-acetylcysteine nor cysteamine blunt the constitutive activation of mTOR in cystinotic cells nor restore the normal equilibrium of mTOR between cell differentiation and cell proliferation [11]. This agrees with the clinical finding that cysteamine does not affect the renal Fanconi Syndrome in cystinosis patients when started after ∼2 years of age [1], but improves GFR and improves, but does not correct, tubular function when started prior to two months of age [15]. Failure of cysteamine may be attributable to the mixed disulfide of cystine and cysteamine only possessing 1 mol of cysteine per mole of disulfide, reducing cysteine delivery by 50 %, and not delivering directly to the mTOR locus as functional cystinosin does.

Elements in cystinosis cell pathology attributable to constitutively active mTOR include AKT, protein kinase C, insulin growth factor receptor (IGF-1R), 4E binding protein 1 (4E-BP1), ribosomal protein S6 kinase (S6K), transcription factor EB (TFEB), sterol-responsive element-binding proteins (SREBPs), Lipin-1, and Unc-51-like autophagy activating kinases. mTOR signaling plays a major role in regulating many critical factors including translation, lipid synthesis, nucleotide synthesis, biogenesis of lysosomes, nutrient sensing, and growth factor signaling [7]. This wide-ranging aberrant cell physiology encompasses much of the cystinosis phenotype (Table 3).

**Table 3:** Elements in cystinosis cell pathology attributable to constitutive mTOR malfunction.

Loss of cell specialization • Create Renal Fanconi Syndrome
Increased apoptosis
Increased autophagy
Aberrant intermediate metabolism • Lipid synthesis (cholesterol) • Nucleotide synthesis • Biogenesis of lysosomes • Nutrient sensing • Growth factor signaling
Aberrant osteocyte development
Failure of proximal renal tubule cells to reabsorb small molecules

Hyperactive apoptosis has been reported in nephropathic cystinosis cells since first described by Thoene in fibroblasts in 2002 [16] and subsequently found to affect proximal renal tubule cells [17]. It was also suggested to be associated with phenotype development [18]. Initially, increased apoptosis in cystinotic cells was attributed to increased activity of PKC∂ after cysteinylation from cystine released from lysosomes [17]. Increased apoptotic activity was found to be associated with AMP kinase [19].

Autophagy is also increased in cystinotic cells [20,21], resulting from mTOR response to the erroneous assessment of starvation state in which cells revert to lysosomal degradation of endogenous proteins, particularly mitochondria, to provide a supply of free amino acids to allow continuation of protein synthesis [7,11].

## 2 Taurine

**Fig. 1:**
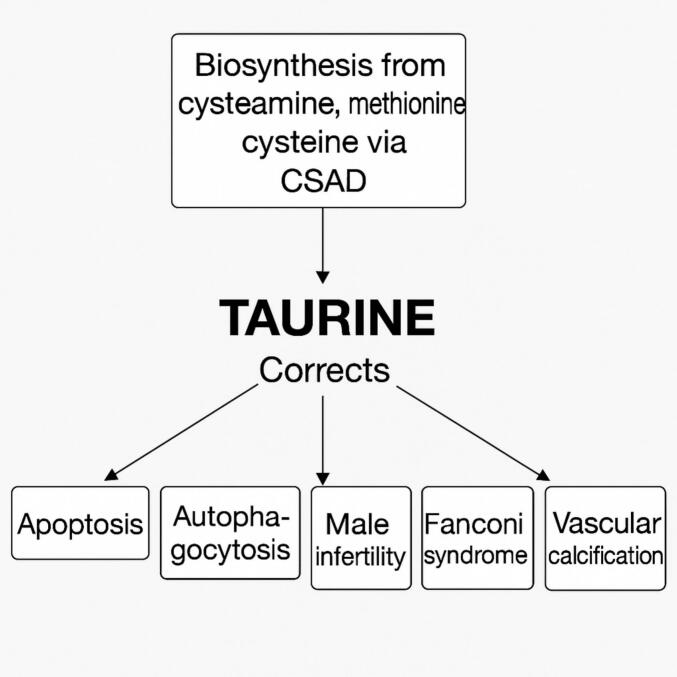
Metabolic source of taurine, and abnormalities taurine has been shown to correct in vitro and in vivo (see Table 3). Abbreviations: Mt - mitochondrial. ROS - Reactive oxygen species. RTE - renal tubule epithelial cells. Tau(T) - Taurine transporter. CSAD - cysteine sulfinic acid decarboxylase.

Taurine (2-aminoethanesulfonic acid), is a zwitterion non-protein forming, non-carboxy amino acid which is synthesized from cysteamine, methionine, and cysteine, (Fig. 1) and is classified as generally recognized as safe by the US FDA and sold in health food stores both in the USA and Europe. Taurine is the most abundant amino acid in mammalian tissue, with significant effects on ion regulation, osmoregulation and cell development [22].

A review of the abnormal elements in cystinosis shows that many of them may be correctable by taurine. A wide variety of cells, tissues and cell pathways in which taurine acts to alleviate these abnormalities are known [[23], [24], [25], [26], [27], [28], [29], [30], [31], [32], [33], [34], [35], [36], [37], [38], [39], [40], [41], [42], [43], [44]] (Table 4). Studies in 13 out of 14 cell or tissue types showed taurine decreased, inhibited, or ameliorated apoptosis. In one, free taurine did not decrease apoptosis, but apoptosis increased when the taurine transporter Tau(T) was knocked out [31]. Taurine suppressed or attenuated increased autophagocytosis in three studies in 3 cell types [[37], [38], [39]]. Other factors improved by taurine include deficient myoblasts [40,41], vascular calcification [42], male infertility [43], and obesity [44] (Fig. 1).

**Table 4:** Summary of Taurine Correction of Aberrant Cell Physiology.

Aberrant physiology	Pathway Effected	Tissue studied	Effect of Taurine	Reference
Increased apoptosis	AKT pathway	Diabetic rat kidney	Ameliorate apoptosis	[23]
	Decreases upregulated Bax and Fas	Streptozotocin treated pancreatic island	Reduced apoptosis	[24]
	Decreased mt permeability	Hypoxic rat retinal ganglion cell	Reduced apoptosis	[25]
	Caspases	Jurkat T-lymphocytes	Inhibit DNA fragmentation	[26]
	Tau(T)	Retinal glial cells	Reduced apoptosis	[27]
	FasL	IL 2 stressed Anti-CD3-activated Jurkat cells	Reduced apoptosis Downregulation of FasL protein	[28]
	Reduced p53 content, and enhanced cellular Bcl-2 content.	Ischemic neonatal mouse cardiomyocytes	resistant to ischemia-induced necrosis and apoptosis.	[29]
	Prevention of apoptosome formation	Ischemia in Cardiomyocytes	Inhibits apoptosis.	[30]
	caspase-3 activity	Cisplatin Erlich ascites cells	No effect of free taurine supplement KO Tau(T) increases apoptosis	[31]
	Reduction in caspase-8 and caspase-9 expression	Ischemic mouse supraoptic and paraventricular nuclei	Reduces apoptosis	[32]
	ROS inhibition and [Ca2+]i stabilization	Glucose exposure to human umbilical vein endothelial cells	Reduces apoptosis	[**33**]
	Prevents p53 activity, ROS generation and Ca2+ mobilization	Doxorubicin exposed rat testis	Reduces apoptosis	[34]
	decreased the expression of ER stress-activated glucose regulatory protein 78, C/EBP homologous protein and caspase-12.	nucleus pulposus cells	Attenuates apoptosis	[35]
	Decreased ROS	Human renal tubules treated with 30 mM glucose	prevents apoptosis	[36]
Increased autophagocytosis	Akt/mTOR	Ca Oxalate exposed renal tubule cells	Suppresses autophagy	[37]
	LC3-II	OTA-exposed PK 15 cells	Attenuated autophagy	[38]
	Attenuates via mTOR pathway	Meth treated PC12 cells	Attenuates autophagy	[39]
Deficient myoblasts	PI3K-ARID4B-mTOR pathway	C2C12 myoblast cells	Promotes proliferation of myoblast cells	**[****40****]**
	Phosphorylated (p-)AKT–p-mTOR	Muscle tissue	Promotes protein synthesis and restoration of functional and structural phenotypes	**[****41****]**
Vascular calcification	mTORC1 activity and endoplasmic reticulum stress-unfolded protein response in uremic vascular calcification.	Mice	Short-term treatment of CKD mice with rapamycin, an inhibitor of mTORC1, or tauroursodeoxycholic acid, a bile acid that restores ER homeostasis, normalized mTORC1 activity, molecular markers of UPR, and calcium content of aortas.	**[****42****]**
Male infertility	Multiple	Multiple	taurine can promote the endocrine function of the hypothalamus-pituitary-testis (HPT) axis, testicular tissue development, spermatogenesis and maturation, delay the aging of testicular structure and function, maintain the homeostasis of the testicular environment, and enhance sexual ability	**[****43****]**
Obesity	Multiple	Multiple	Synthetic activity and concentration of taurine in adipose tissues and plasma have been shown to decrease in humans and animals during the development of obesity, suggesting a relationship between taurine deficiency and obesity	**[****44****]**

Surprisingly, patients with cystinosis not on cysteamine have significantly lower plasma taurine (5.2 ± 3.2 μM) than either cysteamine-treated cystinosis patients (25.1 ± 5.0 μM) or normal controls (109.7 ± 11.8 μM) (Mean ± SE) [45]. In this study the patients included 14 non-cysteamine treated patients with a mean age of 1.04 years, 19 cysteamine-treated patients with mean age of 3.5 years, and 30 normal controls with mean age of 1.44 years.

Why should cystinosis patients have low plasma taurine? The answer is two-fold:

1) Cysteine is the substrate for cysteine sulfinic acid decarboxylase (CSAD), the rate limiting step in taurine synthesis, and the major endogenous source of taurine. Cysteine availability is significantly limited by the defect in cystinosin that precludes delivery of cystine to the cytosol where it is reduced to cysteine and supplies mTOR and CSAD with cysteine [46]. Knockout (KO) mice lacking CSAD have significantly lower taurine concentrations than wild type controls, having a 90 % reduction in hepatic taurine, and 70 % reduction in renal taurine. [47].
2) The Fanconi Syndrome wastes small molecules including aminoacids which are usually retained by the renal proximal tubules, further decreasing the availability of taurine [1].

Although cysteamine therapy raises urinary taurine somewhat [2], it has minimal effect on the renal Fanconi Syndrome, or other phenotypic elements in Table 3. Importantly, supplementation of taurine in the diets of the CSAD (CSD) KO mice normalized solid organ taurine concentration in two to four months [47].

It was recently shown that taurine declines with age in species as diverse as C.

elegans, mice, monkey and man, and taurine supplementation increased the life span of worms, mice, and the health span of aged female mice. Taurine reduced age associated bodyweight gain, improved bone mass in female mice, increased muscle endurance, coordination, and strength [48]. Many elements of the cystinotic phenotype are consistent with premature aging. Premature aging of skin in cystinosis patients has been documented via optical coherence tomography [49]. Bone abnormalities, different than vitamin D responsive rickets [50], male hypogonadism, swallowing abnormalities, vascular calcifications, diabetes, and hypercholesterolemia all occur in cystinosis with a mean age of ∼26 years [6].

In addition to other tissues affected by taurine, the eye has been found to have altered metabolism in ocular tissues [51]. Studies have shown that the retina produces taurine, and CSAD is present in all retinal layers [52]. The salt and pepper retinopathy in cystinosis eyes is one of the first clinical manifestations to appear [1] and is not universally responsive to systemic cysteamine [53]. This retinopathy may be the result of taurine deficiency.

Cystinosis patients have been managed with cysteamine and small molecule replacement but cysteamine's deficiencies, as described above, are a major hindrance to their quality of life. Taurine is benign and may possess the ability to correct these deficiencies. As noted, addition of taurine to the diet of taurine deficient mice raised the taurine content in solid organs to normal in two to four months, supporting the idea that oral taurine will reach the kidneys and other solid organs in cystinotic patients. Since taurine corrects many of the phenotypic abnormalities found in other conditions which occur in cystinosis it appears reasonable to determine its validity via preliminary in vitro and animal studies and a well-controlled clinical trial.

## CRediT authorship contribution statement

**Jess G. Thoene:** Writing – review & editing, Writing – original draft, Conceptualization.

## Declaration of Generative AI and AI-assisted technologies in the writing process

During the preparation of this work the author(s) used [Chat GPT04] in order to produce the figure. After using this tool/service, the author(s) reviewed and edited the content as needed and take(s) full responsibility for the content of the publication.

## Declaration of competing interest

I received no financial support for this work.

I am the inventor of a patent owned by the University of Michigan covering this use of taurine.

I did not use AI in the preparation of this work.

## Data Availability

No data was used for the research described in the article.
